# Pre-hospital suPAR, lactate and CRP measurements for decision-making: a prospective, observational study of patients presenting non-specific complaints

**DOI:** 10.1186/s13049-021-00964-5

**Published:** 2021-10-16

**Authors:** Milla Jousi, Marja Mäkinen, Johanna Kaartinen, Leena Meriläinen, Maaret Castrén

**Affiliations:** 1 Department of Emergency Medicine and Services, University of Helsinki and Helsinki University Hospital, HYKS Akuutti, PL 340, 00029 HUS, Helsinki, Finland; 2 Aidian Oy (Previously Orion Diagnostica), PL 83, 02101 Espoo, Finland

**Keywords:** suPAR, Lactate, CRP, Point-of-care, Emergency medical service

## Abstract

**Background:**

In the pre-hospital setting, non-urgent patients with non-specific chief complaints pose assessment challenges for the emergency medical systems (EMS). Severely ill patients should be identified among these patients, and unnecessary transport to the emergency department (ED) should be avoided. Unnecessary admissions burden EDs, deplete EMS resources and can even be harmful to patients, especially elderly patients. Therefore, tools for facilitating pre-hospital decision-making are needed. They could be based on vital signs or point-of-care laboratory biomarkers. In this study, we examined whether the biomarker soluble urokinase plasminogen activator receptor (suPAR), either alone or combined with C-reactive protein (CRP) and/or lactate, could predict discharge from the ED and act as a pre-hospital support tool for non-conveyance decision-making.

**Methods:**

This was a prospective, observational study of adult patients with normal or near-normal vital signs transported by an EMS to an ED with a code referring to deteriorated general condition. The levels of suPAR, CRP and lactate in the patients’ pre-hospital blood samples were analysed. The values of hospitalized patients were compared to those of discharged patients to determine whether these biomarkers could predict direct discharge from the ED.

**Results:**

A total of 109 patients (median age: 81 years) were included in the study. Of those, 52% were hospitalized and 48% were discharged from the ED. No statistically significant association was found between suPAR and the ED discharge vs hospitalization outcome (OR: 1.04, 95% CI 0.97–1.13, AUROC: 0.58, 95% CI 0.47–0.69). Adding CRP (AUROC: 0.64, 95% CI 0.54–0.75) or lactate (AUROC: 0.60, 95% CI 0.49–0.71) to the regression models did not improve their diagnostic accuracy. None of the patients with a suPAR value of less than 2 ng/ml were admitted to hospital, while 64% of the patients with a suPAR value of more than 6 ng/ml were hospitalized.

**Conclusion:**

Pre-hospital suPAR measurements alone or combined with CRP and/or lactate measurements could not predict the ED discharge or hospital admission of 109 non-urgent EMS patients with non-specific chief complaints and normal or near-normal vital signs.

## Background

Pre-hospital emergency medical system (EMS) units regularly encounter non-urgent patients who have non-specific complaints, such as general weakness and fatigue, a sense of illness, light-headedness and dizzy spells, or are unable to cope with normal daily activities. Patients are often elderly and frail and may have normal or near-normal vital signs. These patients pose assessment challenges for EMS providers [1, 2]. Severely ill patients should be identified among these patients with high precision and certainty. On the other hand, unnecessary transports to emergency departments (ED) should be avoided for patients who will not benefit from them. Such transports can be harmful to patients, especially elderly patients, burden EDs and deplete EMS resources [3–5]. Depending on the local EMS protocols, a patient may stay at home after a proper on-scene evaluation if there is no need for transport to a healthcare facility.

Decision-making in a pre-hospital environment is challenging because of a shortage of information about the patient’s medical history and development of the current complaint [6–8]. Several in-hospital triage tools have been tested in the pre-hospital setting. The vital sign–based triage tool *National Early Warning Score* (NEWS) has been shown to predict early mortality among unselected emergency patients in pre-hospital use [9–11]. Additionally, several biomarkers have been suggested to support decision-making in the pre-hospital environment due to advancements in the point-of-care (POC) laboratory analysis technology.

Lactate is one of the biomarkers that have been evaluated for the assessment of critically ill or trauma patients in the pre-hospital environment [12–14]. Another promising biomarker is the soluble urokinase plasminogen activator receptor (suPAR), the soluble form of the cell membrane–bound protein uPAR. It is released during an inflammatory response or immune activation by immune cells and endothelial cells and can thus reflect the extent of the patient’s immune activation. Low suPAR levels indicate a low risk of critical illness and mortality; in contrast, elevated levels are associated with chronic diseases and severe conditions, such as sepsis or vital organ dysfunctions, and increased mortality [15–17]. A prospective triage study in Denmark found an association between ED suPAR levels and 90-day mortality, higher Charlson Comorbidity Index scores and greater lengths of hospital stay [16]. A large registry-based study (TRIAGE III) investigating suPAR as a risk stratification tool in the ED reported that suPAR measurements improved risk stratification but did not reduce mortality [18]. A study in Italy showed that ED admission suPAR levels were useful for stratification and prediction of 30-day mortality among 130 sepsis patients [19]. ED POC suPAR has also been shown to be a reliable prognostication and triage tool in a limited-resource setting in India, a densely populated country [20]. However, to the authors’ knowledge, there are no published data on the effectiveness of suPAR as an assessment tool in the pre-hospital environment. Therefore, we aimed to examine whether pre-hospital suPAR levels alone or combined with C-reactive protein (CRP) and/or lactate measurements could predict discharge from the ED for patients with non-specific complaints and normal or near-normal vital signs. Since all three biomarkers are available in POC laboratory analyses, they could be used by EMS providers as supporting tools in the non-conveyance decision-making process.

## Methods

This was a prospective observational study that did not affect patient treatment. The patients were recruited between July 2016 and September 2017 by the EMS units of Espoo and Vantaa (Helsinki metropolitan area), which have a total population of 570,000 people and approximately 50,000 annual EMS dispatches. The EMS in organized as a four-tiered system: first responders, basic life support (BLS) units, advanced life support (ALS) units, and medical supervisor units and physician-staffed units. The patients included in the study were assessed by either BLS or ALS units. The dispatch center categorizes the missions into four levels of urgency (A: life-threatening, B: unknown but potentially high-risk, C: urgent but not life-threatening and D: non-urgent but acute situation). Moreover, a code specifying the reason for the mission is provided. After the on-scene assessment of the patient, the paramedics re-categorize the transportation using the same four urgency categories and the specifying codes. If the patient’s main problem is deteriorated general condition with no specific symptoms or findings related to a specified organ dysfunction, a code 774 is used. This code refers to a patient who is conscious and whose condition has deteriorated over a longer period. The units are also allowed to make non-conveyance decisions based on standing orders or after consulting a doctor.

All adult patients transported by an EMS unit to an ED with a code D774 (deteriorated general condition) were screened for eligibility for participation in the study by the treating paramedics. The inclusion criteria were related to the vital signs as follows: systolic blood pressure > 100 mmHg, heart rate 50–119 beats/minute, peripheral oxygen saturation (SpO_2_) > 90% without supplemental oxygen, respiratory rate 10–25 breaths/minute, temperature 36–38.5 °C and Glasgow coma scale score (GCS) 15. Determining the cut-off values was not planned to follow the NEWS criteria but followed expert opinions. They were set before the patient inclusion. All included patients or their next-of-kin provided informed consent before inclusion. The inclusion process and blood sampling did not involve any additional personnel. The paramedics were trained beforehand in the research protocol, patient recruitment and sampling. The study protocol was approved by the Ethics Committee of Helsinki University Hospital (HUS/2495/2017/§222). The reporting of the study followed the ‘Strengthening the Reporting of Observational Studies in Epidemiology’ (STROBE) guidelines for cross-sectional studies.

Blood samples were collected from the included patients in the pre-hospital environment via an intravenous cannula with a Vacuette Holder® (Becton, Dickinson and Company, NJ, USA) in two Vacutainer® tubes (Becton, Dickinson and Company, NJ, USA), one sodium-fluoride/potassium-oxalate tube for lactate and CRP analyses and one EDTA tube for suPAR analyses (4 ml each). The samples were centrifuged in hospital laboratories (HUSLAB). The plasma was frozen within 24 h and stored at − 70 °C for further analyses. The suPAR levels were determined using immunochromatographic rapid tests (suPARnostics® Quick Triage; ViroGates, Birkerød, Denmark) and enzyme-linked immunosorbent assays (ELISA) (suPARnostics® ELISA; ViroGates, Birkerød, Denmark) in the Karolinska university laboratories in Stockholm. The lactate levels were analysed in hospital laboratories (HUSLAB). The CRP measurements were performed using the QuikRead go® CRP test (Aidian Oy, Espoo, Finland), a quantitative POC test for determining CRP in whole blood, plasma, or serum samples. The QuikRead go® instrument performs immunoturbidimetric assays in a measuring range of 5–200 mg/l and provides results in two minutes.

The patients’ data were retrieved from the electronic patient record system of the receiving hospitals (Uranus®; CGI Suomi Oy, Finland) and the pre-hospital electronic EMS records (MerlotMedi®, CGI Suomi Oy, Finland).

The data are expressed as medians (interquartile ranges [IQR]) for continuous variables and counts and percentages for categorical variables. Fisher’s exact test was used for comparisons between categorical variables, and the Mann–Whitney *U* test was used for continuous variables, as they were not normally distributed. Moreover, logistic regression models were used to check whether combining the variables would improve their classification accuracy. The markers’ and models’ performance was assessed using receiving operating characteristic (ROC) curves and areas under the ROC (AUROC) curves. The level of statistical significance was set to *p* < 0.05. The analyses were performed using GraphPad Prism version 9.0 (GraphPad Software, Inc., CA, USA) and R version 4.0.3 (R Foundation for Statistical Computing, Vienna, Austria). A priori sample size calculations were performed only for suPAR measurements. Sample size calculations for the combination of suPAR, lactate and CRP measurements were not performed.

## Results

A total of 230 patients met the inclusion criteria. Thirty patients were excluded because of missing variables, and 91 patients were excluded because of lack of consent. Thus, 109 patients were included in the study. The median age of the patients was 81 years (range: 29–103; IQR: 72–87), and 39% of the patients were male (Table 1). The patients were transferred to the ED by ALS (*n* = 18, 17%) or BLS (*n* = 91, 83%) units. After assessment in the ED, 57 (52%) patients were admitted to a monitored ward (*n* = 1, 1%), hospital wards (*n* = 22, 20%) or primary health care facility wards (*n* = 34, 31%). Fifty-two patients (48%) were discharged either home or to a long-term residential care facility, where they normally lived. The most frequent diagnoses in the ED were malaise and fatigue (*n* = 16), dizziness (*n* = 11), enteritis (*n* = 8), nausea and vomiting (*n* = 5), and unspecified bacterial infection (*n* = 5)*.*

**Table 1:** Vital signs and pre-hospital laboratory values of the patients

	BP	HR	RR	SpO_2_	Temp	NEWS	suPAR	CRP	Lactate
Pre-hospital	144 (168–121)	78 (90–70)	16 (18–15)	96% (98–95)	36.8 (37.2–36.6)	1 (2–0) range: 6–0	4.6 (6.8–3.2)	7 (18–5)	1.8 (2.7–1.3)
Emergency department	141 (163–125)	76 (89–65)	16 (20–15)	97% (98–95)	36.9 (37.1–36.4)	1 (2–0) range: 9–0			

All values are medians (interquartile ranges)

*IQR* interquartile range, *BP syst* systolic blood pressure (mmHg), *ED* emergency department, *HR* heart rate (per minute), *RR* respiratory rate (per minute), *SpO*_*2*_ peripheral oxygen saturation, *Temp* peripheral temperature (°C), *NEWS* national early warning score, *suPAR* soluble urokinase plasminogen activator receptor (ng/ml), *CRP* C-reactive protein (mg/l)

**Fig. 1:**
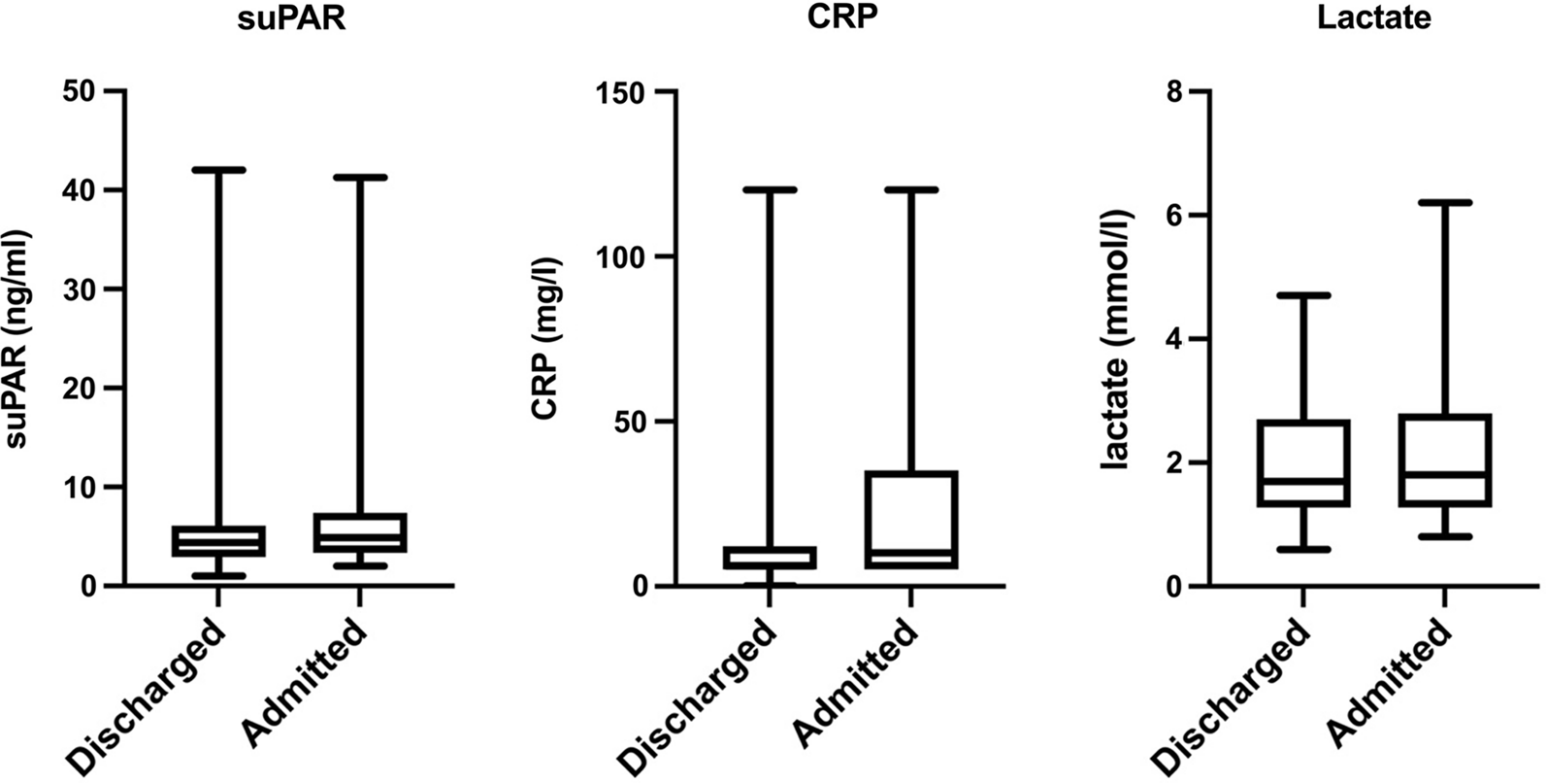
Comparison of suPAR, CRP and lactate values between discharged and hospitalized patients. A two-tailed Mann–Whitney *U* test was used for the comparisons. No statistically significant differences were found between the two groups (suPAR: *p* = 0.132; CRP: *p* = 0.057; lactate: *p* = 0.64). Median (interquartile range) values: suPAR-discharged, 4.4 (6.1–3.0); suPAR-hospitalized, 4.9 (7.4–3.3); CRP-discharged, 6 (12–5); CRP-hospitalized, 10 (35–5); lactate-discharged, 1.7 (2.7–1.3); lactate-hospitalized, 1.8 (2.8–1.3). *suPAR* soluble urokinase plasminogen activator receptor, *CRP* C-reactive protein

**Fig. 2:**
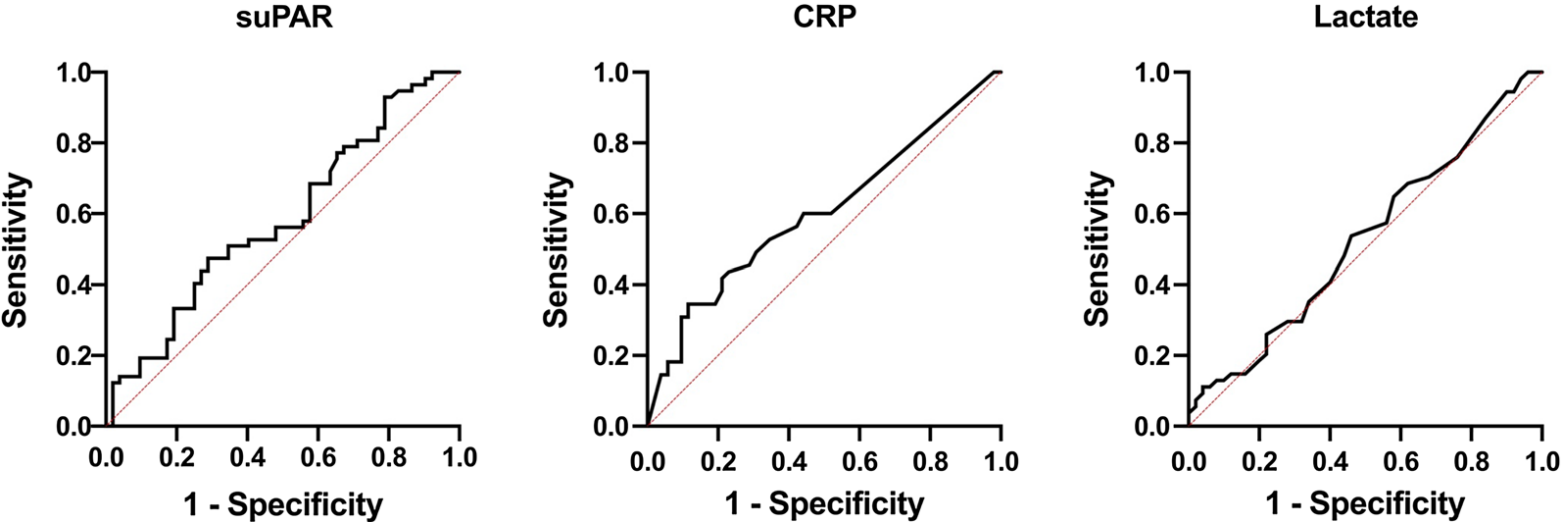
ROC curves of the measured parameters when comparing the patients discharged home from the emergency department with the hospitalized patients. AUROC (95% CI): suPAR, 0.58 (0.48–0.69); CRP, 0.60 (0.50–0.71); lactate 0.53 (0.42–0.64); all statistically and clinically nonsignificant. *ROC* receiver operating characteristic, *AUROC* area under the receiver operating characteristic, *CI* confidence interval, *suPAR* soluble urokinase plasminogen activator receptor, *CRP* C-reactive protein

No statistically significant differences were found between the discharged and hospitalized groups in suPAR, CRP or lactate levels (Fig. 1). Moreover, the ROC curves showed no cut-off values that could accurately distinguish between discharged and hospitalized patients (Fig. 2). The logistic regression model using suPAR and CRP yielded an AUROC of 0.64 (0.54–0.75), whereas the suPAR + lactate model had an AUROC of 0.60 (0.49–0.71). As shown in Table 2, the ORs for suPAR were not significantly far from 1 in either the univariate model or the models combining it with lactate and/or CRP. Even with all three of the variables in the same model, the AUROC was only 0.66 (95% CI 0.56 − 0.77) suggesting that the model did not accurately distinguish between discharged and hospitalized patients.

**Table 2:** Results of the univariate and multivariate logistic regression models. The results are presented as ORs (95% CI) or AUROC (95% CI)

	Univariate	suPAR_lactate	suPAR_CRP	suPAR_CRP_lactate
suPAR	1.04 (0.97–1.13)	1.04 (0.97–1.15)	1.03 (0.96–1.13)	1.04 (0.96–1.15)
lactate	1.13 (0.90–1.63)	1.07 (0.74–1.56)		1.04 (0.71–1.54)
CRP	1.01 (1.00–1.03)*		1.01 (1.00–1.03)	1.01 (1.00–1.03)
AUROC	0.58 (0.48–0.69)^1^	0.60 (0.49–0.71)	0.64 (0.54–0.75)	0.66 (0.56–0.77)

*p < 0.05

^1^AUROC for suPAR univariate model

*OR* odds ratio, *CI* confidence interval, *AUROC* area under receiver operating characteristic, *suPAR* soluble urokinase plasminogen activator receptor, *CRP* C-reactive protein

None of the patients with a suPAR value of less than 2 ng/ml were admitted to hospital. Of the patients with a suPAR value of less than 3 ng/ml, 42% were admitted. Notably, 64% of the patients with a suPAR value of more than 6 ng/ml were hospitalized.

## Discussion

In this population of 109 non-urgent EMS patients (with non-specific chief complaints and normal or near-normal vital signs) transferred to EDs with a code referring to deteriorated general condition, using suPAR levels could not distinguish between discharged and hospitalized patients. Adding CRP or lactate levels to the assessment did not increase the diagnostic accuracy of suPAR for this purpose.

Generally, patients with non-specific chief complaints pose a challenge for EMSs and EDs. In a recent retrospective cohort of 3780 pre-hospital patients with non-specific chief complaints, a serious condition was present in 35% of the patients. The 30-day mortality rate in this group was 20%, compared to 4.2% for patients with no serious conditions [2]. A recent systematic review found that the mortality rate of ED patients with non-specific chief complaints was significantly higher (OR: 2.50) than that of patients with specific complaints [21]. Therefore, it is particularly important for patients with serious underlying conditions to be identified by the EMS, even if they have no specific complaints at the time.

Notably, not all the patients encountered by the EMS need to be transported to hospital. Non-conveyance may be the most appropriate response to patients’ needs. EMS providers may advise patients to monitor the situation at home or to seek non-urgent medical attention. Appropriate care may be provided by family doctors if the paramedics deem the condition non-urgent after careful primary assessment. Besides benefits to the patients, this can reduce the burden on EDs and enhance the performance of EMS services, allowing ambulances to respond more quickly to life-threatening emergencies. The non-conveyance rates vary between countries; in Finland, the rate is approximately 40% [22–24]. The non-conveyance decision-making process is complex and multifactorial [25]. In a recent survey in Finland, higher non-conveyance rates were associated with missions with lower urgency, EMS arrivals during evening and night hours, younger patient ages, female gender, and alcohol use [24]. Wise non-conveyance decisions are beneficial in many respects; therefore, supportive tools for non-conveyance decision-making are needed for this population.

Several vital sign-based triage tools are used in pre-hospital decision-making. The NEWS has been shown to predict early mortality among unselected emergency patients in pre-hospital use [9–11]. The Rapid Emergency Triage and Treatment System-Adult (RETTS-A), which is used in Sweden, and the five-level Taiwan Triage and Acuity Scale (TTAS)have also been tested in the pre-hospital setting and have been found to predict time-sensitive conditions, mortality, and hospitalization. They have also been shown to predict serious conditions among patients with non-specific complaints [2, 26–28].

Various biomarkers have been suggested as tools supporting decision-making in the pre-hospital environment. POC laboratory analysis technology enables on-site measurements with rapid test results. Lactate measurements in the pre-hospital setting have been shown to provide prognostic information superior to that provided by unstable patients’ vital signs [29]. However, the benefits of lactate measurements for non-urgent patients with non-specific complaints remain unknown. Higher levels of suPAR have been shown to predict in-hospital, 30- and 90-day mortality in various groups of patients admitted to the ED, including patients with a low NEWS [16, 30, 31]. A registry-based cohort study of 17,300 patients found that the addition of suPAR measurements to the NEWS significantly improved risk prediction for low- and high-risk patients with acute conditions in EDs [30]. A large clinical trial (TRIAGE III) involving 16,800 patients reported that suPAR measurements in the ED improved risk stratification but did not affect 30-day mortality [18, 32].

Although suPAR has been shown to be associated with several serious conditions, such as sepsis or acute cardiovascular and renal events, in this study, measurements of suPAR did not predict the investigated outcome (discharge from the ED or hospitalization). The studied patient cohort represents an important and challenging patient population that would certainly benefit from supportive tools for decision-making. Unfortunately, this study was unable to prove that suPAR alone or combined with lactate and/or CRP can be such a tool. An important factor to notice is that a discharge directly from the ED is not a synonym for needless ED transport, so the outcome ‘ED discharge/hospital admission’ may not be an ideal substitute for disease severity. Although many conditions, such as infections, may be diagnosed in the ED, the treatment can be ambulatory. Furthermore, the reason for hospital admission may be related to a patient’s social surroundings and may be unpredictable by any biomarker. A pre-hospital suPAR measurement could be considered an additional tool in the EMS toolkit, along with a thorough history taking and examination, supported by a vital sign-based triage tool, such as the NEWS.

This study sample was selected to represent a diagnostically challenging subgroup of patients with non-specific chief complaints and normal or near-normal vital signs. To the authors’ knowledge, this is the first published study to analyse suPAR, CRP and lactate levels collected prospectively in a pre-hospital environment, aimed to identify necessary decision-making tools for a large and challenging pre-hospital patient population. Nevertheless, certain limitations should be noted. Patient selection bias cannot be excluded, as not all eligible patients may have been included since screening was performed by the treating paramedics during their routine work. The number of all eligible patients and the inclusion ratio are unknown. The large number of patients who were excluded due to a lack of consent may also have biased the results. The number of remaining patients may have caused the study to be underpowered. Furthermore, comorbidities were not registered, which may have acted as a confounding factor since elevated suPAR levels are associated with various factors and chronic diseases, such as obesity, physical inactivity, age, malignancies, renal diseases, and cardiovascular diseases. The patients were old (median age: 81 years) which can elevate suPAR levels regardless of the acutely presenting condition. In general, risk assessment models based on vital signs and routine biomarkers, such as suPAR, are less accurate for older patients [33].

This study highlights a relevant risk stratification issue of pre-hospital emergency care among patients with non-specific chief complaints but normal or near-normal vital signs. Some of these patients may benefit from non-conveyance. Due to the difficulty in assessing these patients and the increased mortality rate, accurate tools are needed to support decision-making in the pre-hospital environment to safely leave patients in their homes or advise them to seek non-urgent medical care. Finding a POC laboratory marker that can safely rule out serious conditions as part of a pre-hospital assessment has obvious appeal. However, suPAR alone or combined with lactate and/or CRP was not found to be beneficial in this study.

## Conclusion

Pre-hospital suPAR measurements alone combined with CRP and/or lactate measurements could not predict the ED discharge or hospital admission of 109 non-urgent EMS patients with non-specific chief complaints and normal or near-normal vital signs.

## Data Availability

The data sets used in this study are available from the corresponding author upon reasonable request.

## Abbreviations

AUROC: Area under receiving operating characteristic
BP: Blood pressure
CRP: C-reactive protein
GCS: Glasgow coma score
HR: Heart rate
ED: Emergency department
EMS: Emergency medical system
IQR: Interquartile range
NEWS: National early warning score
POC: Point-of-care
RETTS-A: Rapid emergency triage and treatment system-adult
ROC: Receiving operating characteristic
RR: Respiratory rate
SpO_2_: Peripheral oxygen saturation
STROBE: Strengthening the reporting of observational studies in epidemiology
suPAR: Soluble urokinase plasminogen activator receptor
Temp: Peripheral temperature
TTAS: Taiwan triage and acuity scale
